# IMPACT OF GERIATRIC SCHOLARS PROGRAM PSYCHOLOGY TRACK ACTIVITIES ON VA PSYCHOLOGISTS’ CLINICAL PRACTICE

**DOI:** 10.1093/geroni/igae098.0842

**Published:** 2024-12-31

**Authors:** Christine Gould, Jeffrey Gregg, Rachel Rodriguez

**Affiliations:** VA Palo Alto Healthcare System, Palo Alto, California, United States; Durham VA Health Care System, Durham, North Carolina, United States; Durham VA Health Care System, Durham, North Carolina, United States

## Abstract

The significant workforce shortage for geriatric mental health providers is pronounced within the Department of Veterans Affairs (VA) where more than half of Veterans receiving care are over the age of 65 years. During the past 10 years, the Geriatric Scholars Program-Psychology Track (GSP-P) has developed and delivered a multi-component geropsychology training curriculum for VA psychologists serving older Veterans. Quantitative evaluation findings have demonstrated significant improvements in self-reported geropsychology knowledge and skills. However, detecting changes in clinical practice proves to be more challenging as VA psychologists’ practice includes components that are difficult to quantify (e.g., consultation, environmental interventions, facilitating family and team communication). To characterize GSP-P’s impact on clinical practice, we used a rapid qualitative analysis approach to examine open-ended responses about how clinical practice has changed on a survey of GSP-P alumni (N = 40 respondents, 27% response rate). Most (75%) respondents endorsed that GSP-P activities had an impact specifically on their clinical practice. Scholars noted improved conceptualization of patients’ presenting concerns. They described improved consideration of multiple factors including sensory changes, health literacy, medical health, and family systems. Increased use in standardized assessment measures and assessment of other factors (e.g., medications affecting cognition) emerged as well. Scholars described expanding intervention skills such as applying evidence-based psychotherapies with older adults and involving family members in therapy. These qualitative findings suggest that participation in the GSP-P activities has led to improvements in the quality of care provided to older Veterans with complex mental health, neurocognitive, medical, and functional needs.

